# Determinants and prevalence of relapse among patients with substance use disorders: case of Icyizere Psychotherapeutic Centre

**DOI:** 10.1186/s13011-021-00347-0

**Published:** 2021-02-01

**Authors:** Eric Kabisa, Emmanuel Biracyaza, Jean d’Amour Habagusenga, Aline Umubyeyi

**Affiliations:** 1 Rwanda Palliative Care and Hospice Organization (RPCHO), Kigali, Rwanda; 2 Department of Health Policy, Economics and Management, School of Public Health, College of Medicine and Health Sciences, University of Rwanda, Kigali, Rwanda; 3 Programme of Sociotherapy, Prison Fellowship Rwanda (PFR), Kigali, Rwanda; 4 Department of Epidemiology and Biostatistics, School of Public health, College of Medicine and Health Sciences, Kigali, Rwanda

**Keywords:** Relapse, Substance use disorder, Detoxification‐rehabilitation, Rehabilitation centre

## Abstract

**Background:**

Relapse to substance use after successful detoxication and rehabilitation is a public health concern worldwide. Forty to sixty percent of persons in general relapsed after completing detoxication and rehabilitation treatments. Although substance use remains a burden in Rwanda, very little is known about relapse among people with substance use disorder (SUD). Hence, this study aimed to examine prevalence and the factors associated with relapse to substance use at Icyizere Psychotherapeutic Centre (IPC), Rwanda.

**Methods:**

Retrospective, cross-sectional survey was conducted among 391 patients with SUD at IPC. Multiple logistic regression models using STATA version 13 were used to determine the factors associated with relapse among the patients with SUD.

**Results:**

Majority (84.1 %) of the participants were males. More than half (54.1 %) of them were aged between 18 and 30 years with the age average of 33 years (SD = 11.9 years). The results showed a higher prevalence of relapse among patients with SUD (59.9 %). The multivariate analyses indicated that people with SUD living only with their mothers had a greater risk of relapse compared to those with both biological parents [OR = 1.9, 95 % CI (1.02–3.6), *p = 0.04*]. Patients that were hospitalized between one to three months were more likely (11.2 times) to relapse after treatments compared to those who spent more than three months in hospitalization [OR = 9.2, 95 % CI (1.1–77.6), *p = 0.02*]. Furthermore, people that used more than two substances had 1.5 greater risk to relapse than those who consumed one substance. Participants were more likely to relapse if they lived with their peers [OR = 2.4, 95 % CI: (1.2–7.8), *p = 0.01*] or if they lived in a family with conflicts [OR = 2.1, 95 % CI (1.05–9.7), *p = 0.02*].

**Conclusions:**

This study is conducted at one institution caring for patients with SUD. The prevalence was 59.9 %. Future studies are recommended to investigate the effectiveness of the existing relapse prevention programs in order to adjust prevention strategies.

## Background

Relapse refers to a breakdown in the person’s attempt to change substance use behaviors or return to pre-treatment levels of drinking or continue using substances after a period of sobriety or setback in a person’s attempt to change or modify any target behavior [1, 2]. The substance use after successful detoxication and rehabilitation is a common problem globally and it remains higher in low and middle income countries (LMICs) than the high income countries [3, 4]. Diagnostic and Statistical Manual of Mental Disorders (DSM-5) states that substance use disorder (SUD) are characterized by maladaptive patterns of substance use leading to clinically significant impairment or distress [5, 6]. The evidence coming from recent robust epidemiological studies have showed that substance use after successful treatment and rehabilitation is the biggest problem that requires effective preventive measures. More than 50 % of person with SUD relapsed after treatment [4, 6]. Other studies have documented that the relapse rates following treatments are high [4, 7] and typically reaches 40–75 % in 3 weeks to 6 months period following treatment [6, 8–10].

The studies conducted in different countries with high rates of completion of inpatient treatments show a high prevalence of relapse with 33 % in Nepal, 55.8 % in China and 60 % in Switzerland. Similar studies find out that the relapse is between 1 month and 1 year after discharge from treatment programs [6, 10, 11]. From the treatment perspective, relapse and recovery is key issues, highly prevalent and frustrating problem, although the prevalence of patients with substance use remain high [12, 13]. Relapse is the health concern that can be triggered by stress, cues associated with past drug usage, or re-exposure to the substance [5, 14].

Substance use dependence are episodic, with periods of abstinence, reduction of use, and relapse the prevailing pattern, often with the course of events being influenced by external factors such as availability of drugs and societal pressures [9, 15]. Moreover, various factors are associated with relapse to SUD. Those factors are classified into individual, socio-demographic, psychiatric, medical conditions, and socio-cultural influences that may be controlled [16–18]. For instance, preceding studies found that young age at initiation, sex, unemployment, singular status, peer group influence, family history of substance use, conflict and poor family support, environmental factors like availability and accessibility of drugs are contributing factors of relapse [2, 7, 9, 19–22]. Physical factors can also increase the risk of relapse including physical illnesses, physical dependence on drugs, withdrawal from drugs and being in negative physical state [20, 23, 24]. In addition, a previous study showed that 50 % of old friends influenced people with addiction to get the habit to retake drugs after discharge from rehabilitation centres [6, 21, 25]. It was also reported that people with SUD who have been discharged from rehabilitation centres and living in drug-free social environment had higher abstinence rates and are low in relapse to SUD [8, 19, 26]. On the other hand, multiple factors such as post-treatment incarceration, mental or other co-morbid disorders, craving for drugs were reportedly associated with relapse [7, 10, 22].

The Rwanda Mental health survey conducted in 2017 reported that the prevalence of SUD in population aged 14–65 years has been 0.4 % for males and 0.2 % for females [27, 28], further it was demonstrated that the population aged 46–55 years had the highest prevalence (0.6 %) compared to other age groups. Little research is available on relapse following SUD treatment in Rwanda. This leads to poor relapse prevention strategies in different rehabilitation centresers. After realizing that the prevalence of SUD is high especially in youth and most of people treated in those centre are often the same due to relapse, we decided to conduct this study to show its prevalence and the reasons of relapse. The rationale of this study is that the findings will provide knowledge as baseline for other researchers to carry out the similar or related studies countrywide. The findings will also contribute to setting up preventive strategies for reducing the relapse on substance use and manage its risk factors. We hypothesized that SUD would increase the probability of a high prevalence of relapse among patients with SUD and that socio-demographic characteristics would constitute independent risk factors for the relapse in the targeted setting for this research.

### Conceptual framework

**Fig. 1:**
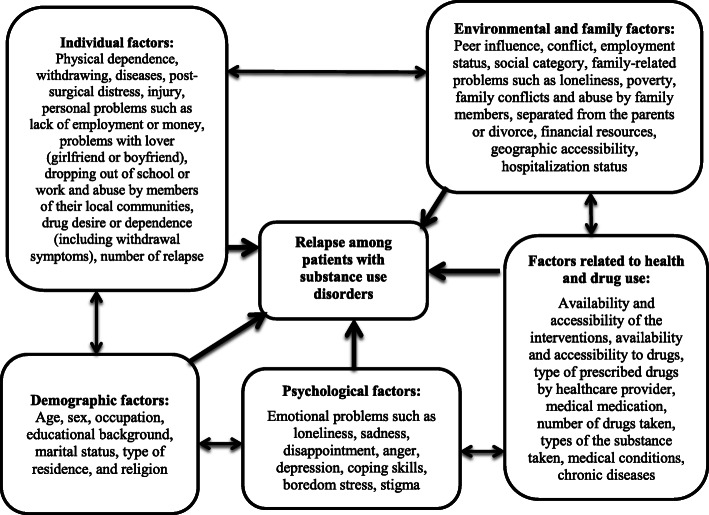
Conceptual framework. Based on the prior studies, this figure shows that the relapse is associated with various determinants that are classified into environmental factors, interpersonal or social factors, socio-demographic factors, intrapersonal factors and physical factors. Environmental included the factors such as availability and accessibility to drugs, interpersonal or social factors include peer influence, social category, conflict, employment status. But socio-demographic characteristics include the factors such as age, sex, residence, education, marital status, parental status of the participants. For intrapersonal factors, prior studies reported negative emotion whereas phsyicla factors included chronic or acute diseases, physical dependence, withdrawal, illness, post-surgical distress and injury

Figure 1. displays the tentative concepts and several ideas that result in a unified conceptual framework and then leads to unified holistic understanding of the phenomenon - relapse and its factors - under the present study. This conceptual framework indicates various factors classified into socio-demographic, environmental, interpersonal, intrapersonal and physical risk factors. This framework was designed using the literature from the prior studies.

## Methods

### Study design

Retrospective cross-sectional survey was conducted to assess the prevalence and contributing risk factors to relapse among patient with SUD at IPC. The study was conducted using secondary data from patient’s records in five consecutive years from 2014 to 2018.

### Study setting, participants and sampling

The study was conducted at Icyizere Psychotherapeutic Centre (IPC) located in Kicukiro District, Niboye Sector, Kigali City, Rwanda. Icyizere Rehabilitation centre is a branch of CARAES-Ndera Neuro-psychiatric Hospital that has specialized unity in drug-related. This Rehabilitation Centre is one of three detoxication and rehabilitation centres for patients with SUD in Rwanda including Iwawa rehabilitation centre located in Western Province and Isange Rehabilitation Centre located in Huye district, Southern Province, Rwanda. These rehabilitation facilities provide various treatments and psychosocial interventions for patients with SUD. IPC is a mental health centre that offers detoxication and rehabilitation for patients with SUD or with comorbidity of psychiatric illnesses. The mission of the three centres is to rehabilitate delinquents from across the country so that they stop drug use or any other deviant behavior. These delinquents are equipped with vocational and hands on skills that help them integrate in the community and participate on the socio-economic and political development. A variable indicating co-morbid psychiatric disorder (0 = no, 1 = yes) was based on recorded International Classification of Diseases (ICD-10) diagnoses (F20–F99), which was either registered during a previous mental health or SUD treatment stay, and/or based on the clinicians’ assessments in the period between 2014 and 2018. These patients are treated as in or outpatients. At this centre, especially in the unit of detoxication and rehabilitation, 1135 patients with SUD were treated in 2017, while in 2018 were 966 both Outpatient Department (OPD) and inpatients.

Participants to this study included hospitalized patients who were admitted due to SUD at IPC between 2014 and 2018 and found in patient’s register and medical files in that period. We have excluded patients who were not hospitalized in the period of five consecutive years (2014–2018), outpatients and patients files which were not well completed (addresses and diagnosis not visible). However, no medical registers or register were excluded because there was no missing variable for the patients diagnosed with SUD. At the end, all medical patients’ files (*n* = 391) identified through the registers from 2014 to 2018 were considered.

### Variables of the study

#### Outcome variable

The outcome variable for this study was relapse among patient with SUD. This variable was binary; the relapse case was the patient who was previously treated, rehabilitated for the SUD and discharged but after a certain period; the same patient was rehospitalised for the same diagnosis. To confirm relapse case, the medical tests including urine toxicology, blood test, and alcohol test were measured at the discharge time and when the patient came back for the same reason, the same exams were taken to confirm the relapse. By checking the medical records, the relapse cases were mentioned after finding the positive results from the urine, blood and alcohol tests. Therefore, in this study, relapse was dichotomized into being relapsed, as opposed to not being relapsed.

### Explanatory variables

The independent variables were collected using medical records and registers. Socio-demographic variables that were used include age, gender, marital status, education, residence, religion, occupation, and parental status. The environmental variables included were the availability, the accessibility to drugs and the types of accessibility. Further, peer group pressure and social problems were also included. The physical factors considered physical dependence, withdrawal, chronic and acute illnesses, post-surgical distress and injury. In addition to these variables, we included the variables such as hospitalization time, substance used. Furthermore, the research included the variables such as stressful influences, family related stressors, psychiatric illness and drug usage, medical conditions and drugs, type of medical condition. The consideration of all independent variables was based on their relationship with relapse demonstrated in prior studies [29, 30].

### Data collection

Data collection was performed by the first author and two trained data collectors whose background is clinical psychology. To collect accurate data and become experts in the research tool, data collectors were provided with short term training about the questionnaires and how to use the medical records of the patients with SUD.

### Data analysis

STATA version 13 was used to perform descriptive and analytical analyses. For descriptive statistical analysis, prevalence of relapse related SUD was computed. The characteristics of the participants were presented using frequency and percentage. For analytical analysis, bivariate and multivariate logistic analysis techniques were used to determine the association between explanatory variables and relapse. Variables that indicated *p* < 0.05 in bivariate analyses were included in the multivariate logistic regression model. This was because the significance level of 5 % was considered sufficient to control residual confounding in the final multivariable model. The odds ratio was computed for indicating the association between relapse and explanatory variables within 95 % confidence intervals and 5 % significance level. Therefore, in this study, the guidelines for strengthening the Reporting of Observational Studies in Epidemiology statement in writing the manuscript (STROBE) was respected [29].

### Ethical consideration

In accordance with the Declaration of Helsinki [30], before using patients medical files, the IPC was contacted to provide the permission to use the information related to this study available in patient’s files and registers . Then, the authorization to access patients data admitted at IPC, a branch of CARAES Ndera Neuro-psychiatric hospital was obtained from the Ethical Committee of CARAES-Ndera Neuro-psychiatric Hospital N°: (007/CNEC/2019). The study protocol was reviewed and approved by Institutional Review Board of College of Medicine and Health Sciences (IRB/CMHS) within the University of Rwanda (No 307/CMHS IRB/2019). Data collection forms were coded and anonymity was respected for confidentiality purpose. After the data collection, all forms were kept in a locked cupboard and only the principal investigator has access to the key.

## Results

### Prevalence and socio‐demographic characteristics

Table 1. demonstrates the description of the participants using the frequencies and percentage. Out of 391 patients with SUD, 62(15.9 %) were females and 329(84.1 %) were males. Concerning age, the mean age was 33 years (SD = 11.8 years). Majority (54.2 %) of the patients was aged between 18 and 30 years and in terms of marital status, majority (78.8 %) was males and single. Concerning education, majority (53.5 % )of the patients did secondary education. In terms of occupation, majority (47.3%) were student while  (26.6 %) did agricultural or no ocupation . By looking at religious status, majority (59.3 %) was Catholics and minority (2.5 %) was with no any religion. Nearly 78 % were living in the city of Kigali. Concerning the prevalence of relapse, the results indicated that 59.9 % of the participants had relapsed one or more time after being discharged from the treatment centre and most of them were relapsed one to two times (52.7 %).

**Table 1:** Prevalence and socio-demographic characteristics of participants (*N* = 391)

Characteristics	Frequencies (*n* = 391)	Percentage
**Relapse**		
No	157	40.1
Yes	234	59.9
**Number of relapses**		
None	157	40.1
One/Two	206	52.7
Three and above	28	7.2
**Participants’ age**		
18–30	212	54.2
31–40	88	22.5
41–50	57	14.6
51–81	34	8.7
**Gender**		
Female	62	15.9
Male	329	84.1
**Marital status**		
Married/cohabiting	70	17.9
Separated/Widow/Divorced	13	3.3
Single	308	78.8
**Education**		
Less than secondary	21	5.4
Secondary	209	53.5
University	161	41.1
**Occupation**		
No occupation/Agriculture work	104	26.6
Professional	75	19.2
Sales services/Domestic work	27	6.9
Student	185	47.3
**Religion**		
Catholic	232	59.3
Muslim	10	2.6
No Religion	10	2.6
Protestant/Adventist	139	35.5
**Residence**		
Kigali city	305	78
Eastern Province	26	6.6
Foreigners	20	5.1
Northern Province	6	1.5
Southern Province	26	6.7
Western Province	8	2.1
**Parental status**		
All alive	207	52.9
No parents	91	23.3
Only father alive	17	4.4
Only mother alive	76	19.4

The table displays the socio-demographic characteristics of the participants and then described them using the frequencies (*N* = 391) and percentages. In this table, the prevalence of the relapse was indicated as the main outcome of this study

Concerning education: The participants who attended primary school were coded as “1”, who attended secondary school were coded as “2” while the highest level of education was University of higher that was coded as “3”. All participants attended schools and had different level of education

### Description of factors influencing relapse in SUD

Table 2 shows the description of factors influencing relapse in substance use disorders. The results found that 98.2 % of the total study population was hospitalized between one and three months and only 1.8 % was followed in hospitalization between two and twelve months. A big number used two to three substances 54.2 %. Peer group influence was high with 81.1 %. Results documented that 70.1 % the patients were influenced by the accessibility of substances including 55 % who had barriers to financial and geographical accessibility. We have also found that stressful influence was another factor that influences SUD at 76 %. Results indicated that the family conflict was 39.1 % followed by inoccupation with 20.2 %. Family conflict combined with other stressful events like poverty, inoccupation and inability to pay loans was 13.3 %. A low percentage of patients with psychiatric illness combined with substance use were reported. 16.9 % of them had at least one psychiatric illness such as bipolar mood disorder, psychosis, posttraumatic stress disorders (PTSD), and depression whereas 0.8 % had two psychiatric illnesses and more psychological disease like depression, psychosis and PTSD. The participants with other medical conditions were 3.6 % whilst psychiatric diseases and acute diseases were 2.8 % and 0.8 % respectively.

**Table 2:** Description of factors influencing relapse in substance use disorders (*N* = 391)

Variables	Frequency (*n* = 391)	Percent
**Hospitalization time**		
Between one and three months	384	98.2
Between three and twelve months	7	1.8
**Substance used**		
Four substances and above	44	11.3
One substance	135	34.5
Two/Three substances	212	54.2
**Peer groups**		
No	74	18.9
Yes	317	81.1
**Accessibility**		
No	117	29.9
Yes	274	70.1
**Type of accessibility**		
Financial accessibility	6	1.5
Geographical accessibility	53	13.6
Accessibility & financial accessibility	215	55
None	117	29.9
**Stressful influences**		
No	94	24
Yes	297	76
**Family related stressors**		
Family conflict	153	39.1
Poverty	52	13.3
Inoccupation	79	20.2
None	94	24
Others (poor insight, poor management of triggers,…)	13	3.3
**Psychiatric illness and drug use**		
No	322	82.3
Yes	69	17.7
**Medical conditions and drugs**		
No	377	96.4
Yes	14	3.6
**Type of medical condition**		
Acute diseases (Gastritis, Pancreatic)	3	0.8
Chronic diseases (Diabetes, Cancer, Hypertension, Hepatitis, HIV/AIDS)	11	2.8
None	377	96.4

The table displays the descriptive analysis for the medical and clinical characteristics. The hospitalization time had two categories (1 to 3 months; 3 to 12 months of stay at the heath facility), type of medical conditions, the patients were categorised into acute diseases (gastritis and pancreatic), chronic diseases (Diabetes, Cancer, hypertension, hepatitis or HIV), and the third category was for the patients with no chronic diseases. The other characteristic was for the patients with stress that was coded as 0 = no stress and 1 = Stress. The families influenced were analysed where 1 represents family conflicts, 2 = poverty, 3 = unemployment, 3 = no family influence, and 5 = other family influence such as poor insight, or poor management of triggers.; *HIV*: Human Immunodeficiency Virus/ Acquired Immuno-Deficiency Syndrome

### Association between relapse and socio‐economic profiles

Table 3 indicates the results of bivariate logistic linear regression analyses corresponding to the factors associated with relapse among patients with SUD. Statistically, significant factors associated with relapse included parental status, hospitalization (*p* = 0.01), hospitalization time (*p* = 0.01), number of different substances used (*p* = 0.008), peer pressure (*p* = 0.001), psychological stress (*p* < 0.001), type of accessibility (*p* < 0.001), number of substances, peer group influence, stressful events, family conflict, others stressful events and inoccupation. But relapse was not significantly associated with age (*p* = 0.6), gender (*p* = 0.9), marital status (*p* = 0.7), residential setting (*p* = 0.3), education level (*p* = 0.1), religion (*p* = 0.2), occupation status (*p* = 0.8), psychiatric diseases, accessibility (*p* = 0.07), medical conditions (*p* = 0.8) and type of medical conditions (*p* = 0.9).

**Table 3:** Bivariate analysis of relapse among patients with substance use disorders (*N* = 391)

Characteristics	Relapse			Odds ratio		
	**Yes**	**No**	**Total**	**OR**	**95 % CI**	***p-value***
**Age of the respondents**						0.6
18–30	130	82	212	**Ref**.		.
31–40	52	36	88	1.1	(0.6–1.8)	
41–50	35	22	57	0.9	(0.5–1.8)	
51–81	17	17	34	1.5	(0.7–3.2)	
**Sex**						0.9
Female	37	25	62	**Ref**.		
Male	197	132	329	0.9	(0.5–1.7)	
**Marital Status**						0.7
Married/Cohabiting	41	29	70	**Ref**.		
Single	184	124	308	0.9	(0.5–1.6)	
Separated/Widow/Divorced	9	4	13	0.6	(0.17–2.2)	
**Level of education**						0.1
Less than secondary	11	10	21	**Ref**.		
Secondary level	117	92	209	0.8	(0.3–2.1)	
University level	106	55	161	0.5	(0.2–1.4)	
**Occupation**						0.8
No occupation/agriculture work	60	44	104	**Ref**.		
Professional	44	31	75	0.9	(0.5–1.7)	
Sales services/ domestic work	15	12	27	1.1	(0.4–2.5)	
Student	115	70	185	0.8	(0.5–1.3)	
**Religion**						0.2
Catholic	146	86	232	**Ref**.		
Protestant/Adventist	77	62	139	1.3	(0.8–2.1)	
Muslim	7	3	10	0.7	(0.1–2.8)	
No Religion	4	6	10	2.5	(0.6–9.2)	
**Residence**						0.3
City of Kigali	186	119	305	**Ref**.		
Eastern Province	14	12	26	1.3	(0.5 2.9)	
Northern Province	5	1	6	0.3	(0.03–2.7)	
Southern Province	17	9	26	0.8	(0.3–1.9)	
Western Province	4	4	8	1.5	(0.3–6.3)	
Foreigners	8	12	20	2.3	(0.9–5.9)	
**Parental status**						0.01**
All alive	129	78	207	**Ref**.		
No Parents	59	32	91	0.8	(0.5–1.4)	
Only mother alive	37	39	76	1.7	(1.02–2.9)	
Only father alive	9	8	17	1.4	(0.5–3.9)	
**Hospitalization time**						0.01**
Between three and twelve months	1	6	7	**Ref**.		
Between one and three months	233	151	384	9.2	(1.1–77.6)	
**Number different substances used**						0.008**
One substance	73	62	135	**Ref**.		
Two/three substances	141	71	212	0.5	(0.3–0.9)	
Four substances and above	20	24	44	1.4	(0.7–2.7)	
**Peer group pressure**						< 0.001*
No	31	43	74	**Ref**.		
Yes	203	114	317	0.4	(0.2–0.6)	
**Accessibility**						0.07
No	62	55	117	**Ref**.		
Yes	172	102	274	0.6	(0.4–1.03)	
**Type of accessibility**						0.03**
None	62	55	117	**Ref**.		
Geographical/financial accessibility	136	79	215	0.6	(0.4–1.03)	
Geographical accessibility	35	18	53	0.5	(0.2–1.1)	
Financial accessibility	1	5	6	5.6	(0.6–49.7)	
**Stressful influence**						< 0.001*
No	28	66	94	**Ref**.		
Yes	206	91	297	0.1	(0.1–0.3)	
**Social problems (stress)**						< 0.001*
None	28	66	94	**Ref**.		
Poverty	30	22	52	0.3	(0.1–0.6)	
Family conflict	124	29	153	2.1	(1.1–7.1)	
Inoccupation	48	33	81	0.2	(0.1–0.5)	
Others	4	7	11	0.7	(0.2–2.7)	
**Psychiatric illness**						0.3
No	191	131	322	**Ref**.		
Yes	43	26	69	0.9	(0.5–1.6)	
**Medical condition**						0.8
No	226	151	377	**Ref**.		
Yes	8	6	14	1.1	(0.3–3.3)	
**Type of medical condition**						0.9
None	226	151	377	**Ref**.		
Chronic diseases	6	5	11	1.2	(0.3–4.1)	
Acute diseases	**2**	**1**	**3**	0.7	(0.1–8.3)	

This table indicates a bivariate logistic regression analyses for determining the association between relapse and socio-demographic characteristics and medical factors collected from the medical files of the admitted patients at Icyizere Rehabilitation Centre. The confidence intervals (95 %) were computed. Thus, statistically significant results when using odds ratio corrected p-values at 0.05 and 0.01.

(*) indicates significant level at 1 % and (**) at 5 %; *Ref*: Reference group or comparable group; *OR*: Odds ratio

### Risk factors of relapse among the person with SUD

Table 4 indicates multiple logistic regression models that illustrate variables related to the likelihood of relapse among the patients with substance use disorders at Icyizere Rehabilitation Centre. The current model included all the variables that were statistically significant at 5 % or 1 % in the bivariate logistic regression. So, it include parental status, hospitalization time, influences of peer groups, accessibility, types of accessibility, number of substances used, influences of stress, and family related problems. The results from the model showed that the most significant common factors of relapse were peer group influence and family-related stresses. The patient with SUD and lived with only their mothers had a greater risk to relapse than those living with both biological parents [OR = 1.9, 95 % CI (1.02–3.6), *p* = 0.04]. The patients who spent one and three months of hospitalization were 11.2 times likely to relapse after treatments compared those hospitalized more than three months of hospitalization [OR = 9.2, 95 % CI (1.1–77.6), *p* = 0.02]. Additionally, findings indicated that patients used two to three substances were associated with increase of odds of relapse compared to those using substance [OR = 1.5, 95 % CI(1.3–8.9), *p* = 0.02]. Participants were more likely to relapse if they were living with their peer or drug dealers [OR = 2.4, 95 % CI (1.2–7.8), *p* = 0.01], family related problems such as intra-family conflicts [OR = 2.1, 95 % CI(1.05–9.7), p = 0.02]. But the results indicated that the participants with geographical accessibility had less risk to relapse than others [OR = 0.09, 95 % CI (0.008-0.9), *p* = 0.04] (Table 4).

**Table 4:** Multivariate logistic regression analyses estimating the risk factors of relapse (*N* = 391)

Variables	Odds ratio	95 % CI	*P. value*
**Parental status**			
All parents alive	**Ref**.		
No Parents	0.8	(0.4–1.5)	0.6
Only mother alive	1.9	(1.02–3.6)	0.04**
Only father alive	1.5	(0.5–4.8)	0.3
**Hospitalization time**			
Between three and twelve months	**Ref**.		
Between one and three months	11.2	(1.1–105.5)	0.02**
**Substance used**			
One substance	**Ref.**		
Two/Three substances	1.5	(1.3–8.9)	0.02**
Four substances and above	1.6	(0.7–3.6)	0.2
**Influences of peer group**			
No	**Ref**.		
Yes	2.4	(1.2–7.8)	0.01**
**Accessibility**			
No	**Ref**.		
Yes	6.1	(0.5–64.4)	0.1
**Type of accessibility**			
None	**Ref**.		
Geographical accessibility, financial accessibility	0.1	(0.01–1.1)	0.06
Geographical accessibility	0.09	(0.008–0.9)	0.04**
**Stressful influence**			
No	**Ref**.		
Yes	0.3	(0.1–1.6)	0.2
**Family related problems**			
None	**Ref**.		
Poverty	0.6	(0.1–2.5)	0.4
Family conflict	2.1	(1.05–9.7)	0.02**
Inoccupation	0.6	(0.1–2.4)	0.5

The table displays a multiple logistic regression model analyses for indicating the determinants of relapse among the patients at the Icyizere rehabilitation centre which is for patients under treatments of substance use disorders (SUDs). The model include: parental status, hospitalization, number of substances used, types of accessibility, accessibility, stressful influences, and family related problems

The 95 % of the confidence interventions and the significance levels at 0.05 and 0.01 were applied.

* Statistically significant at 0.05, ** Statistically significant at 0.01; *CI*: Confidence Intervals; *OR*: Odd ratio

## Discussion

This study explored the prevalence and risk factors of the relapse among the patients with SUD who were hospitalized at IPC of Rwanda. Majority (54.2 %) of the participants was aged 18–30 years and 84.1 % were males. Different studies also documented that SUD mostly occur among younger people than older age people who can maintain abstinence [31, 32].

The findings of this study reported that approximately 58 % of persons relapse between two weeks and three months respectively following treatment for substance use, and as high as 90 % when relapse has been defined as the consumption of a single drink after treatment. These results are consistent to the previous studies that indicated that the average time from abstinence to relapse varies from 4 to 32 days for tobacco, alcohol, and opiates [2, 5, 11, 33]. They are also supported by the prior studies that showed that substance use following treatment typically is higher up to more than 75 % in the 3-6-month period following treatment [30, 34]. Considering South African statistics in 2013, 22 % of admissions into treatment centres were relapsed. These findings provide some insights into the significance of our results that revealed that more than one in two patients (rate of 59.9 %) was relapsed one or more times after completing the treatment.

The results revealed that there was a significant association between parental status and relapse where an orphan of father has a significant greater risk to relapse after treatment. Numerous studies have shown that the parent’s primary role during treatment is to provide support, and in some cases, this involves treatment for the parent directly [35]. In fact, most rehabilitation centre reported that it is often the mother or father who initiates treatment for their minor or adult children. Drug addiction is such a severe condition that evens the strongest-willed individuals cannot get help on their own. Patients with SUD, therefore, are dependent upon their parents to reach out for help for them that contribute to their health improvement. But the role of parent goes far beyond just getting the patients with SUD to treatment. Several studies found that the absence of parents or poor parent-child attachment especially a father makes the children to be independent and sometimes they do not obey the instructions of their mother. For the young people with SUD; this can lead to relapse after treatment due to poor support and follow-up by the family [5, 11, 36].

Our findings revealed that hospitalization time was also significantly associated to relapse where those who are admitted one to three months had greater risk to relapse comparing to SUD than those who were hospitalized more than three months. The length of stay (LOS) at IPC for patients with SUD also is a commonly debated subject amongst treatment professionals. Majority of studies examining LOS on abstinence rates post treatment find a direct relationship between longer duration of stay and higher abstinence rates. In a study solely examining patient in residential treatment, abstinence rates were lower for those who had short stays (1–30 days) than those who had longer LOS. Similarly, individuals who attended a long term institutional treatment were found to be more likely to be abstinent at six months post-treatment compared to the short-term (three to six month) residential treatment program [35, 36]. Moreover, the results of this study suggest that the PUD who experienced low accessibility to drugs had a greater risk to relapse than the patients who appropriately accessed to required health treatments. This result is in line with the previous studies that confirmed that environmental risk factors increase the risk of relapse such as the increased availability and accessibility of drugs [19, 21, 34, 35, 37].

The results revealed that patients using multiple substances such as two to three substances were more likely to relapse than those only used one substance. These results collaborated with the prior studies [17, 23, 37, 38]. Indeed, those using four substances and above were not significant but we suppose that is due to small sample size. Our findings are also supported by the prior studies that found that those exposed to single substance had longest abstinence period than those consumed multiple substances [13, 31, 32]. Peer group was also found to be associated with relapse. Peer influence is a complex issue due to the potentially wide variety of contexts involving friendships and social networks. A number of studies have addressed peer groups influence and have contributed to the understanding of the structure and size of such networks and their association with relapse to SUD. The previous studies found 50 % of old friends influenced former patients with SUD to pick up the drug taking habit after they were discharged from rehabilitation centres [6, 39]. The same author also showed that 76 % of the old friends assist rehabilitated individuals to get the needed supply of drugs. It was indicated that the social context can serve either as a resource or an obstacle for behavior change by the patient post treatment. For example, “negative peer influences have been noted in the development of substance use behavior and the promotion of relapse [9, 33]. Similar authors found that patients were unable to resist either direct or indirect attempts by others to engage them in drinking. Further, they added that the culture makes the substance the recreational drug of choice, it is difficult for the patient to maintain abstinence [3, 19]. The results of this study highlighted that the patients from the families that experience family conflicts were more likely to relapse to substance use compared to the PWUD from the families that have not family conflicts. These results also showed that the family conflicts also may reinforce contradictions between parents, poor parenting skills and an inadequate monitoring style. These results were supported by the preceding studies that confirmed that peer groups and domestic violence are the risk factors of relapse to substances among the patients with SUD [20].

Furthermore, the results revealed that the risk factors of relapse after undergoing the treatment process are lack of living with only mother, few time for hospitalization such as between one and three months, consuming two to three substances use, peer pressure, accessibility to drugs, family and social related problems such as family conflicts. These results are relevant to the prior studies that indicated that the major causes of relapse after undergoing the treatment process include lack of self-confidence, peer pressure, inability to give old habit, easy access of drugs, lack of family and social acceptance and family and social adjustment problems [8, 11, 12, 16, 18, 40].

### Strengths and limitations

The current research had important contributions to the CARAES Ndera Neuropsychiatric Hospital and especially IPC that tend to focus on patients with SUD treatments. It can be also a baseline for other researchers who want to contribute in prevention of relapse among patients with SUD. Data from 2014 to 2018 were pooled together to create large sample size of patients with SUD. The materials used were standardized and no missing variables were found from the medical records. This increased the validity and reliability of the findings from this study. However, some limitations were found in this scientific work. Due to limited financial resources and time, this research was focused on one detoxication and rehabilitation centre while there are other centres in the country. Secondly, as the study was retrospective cross-sectional design, it was limited to available  data, therefore some variables (e.g. household wealth index, medication) that were found in the literature were not collected in the medical records. Furthermore, the study was limited to the target population that did not permit the researchers to generalize at the national level . This study was limited to the cross-sectional design that did not allow the researchers to provide pertinent conclusion about causality of the factors in which we examined. 

## Conclusions

The prevalence of relapse after treatment of SUD was high and the risk factors identified included family conflicts, psychological stress, peer influence and socio-economic status such as availability and accessibility of drugs, peer group influences and lack of assertiveness. Thus, the substance use management should not be limited to detoxification only but emphasis should be given on longer follow up in order to prevent relapse.

IPC was recommended to add in their annual reports the situation of relapse. It was also recommended to set-up relapse prevention measures of their patients after discharge. The Ministry of Health was recommended to evaluate the effectiveness of existing relapse prevention strategies and how they are implemented; to set a consistent program of relapse prevention in order to reduce the high prevalence of relapse; and organize several sensitization campaigns for awareness of the burden of substance use, relapse after treatment and its impacts to the people’s health but also the community. The further studies should be carried out on the prevalence of relapse and factors associated to substance use disorders at the national level.

## Data Availability

The datasets used and analyzed in the current research are available from the corresponding author on researchable request. Although all data analyzed are included within this article, they may be shared when necessary but also data collection forms.

## Abbreviations

CARAES: Cartate Aergrorum Served
DSM: Diagnostic and Statistical Manual of Mental Disorders
IPC: Icyizere Psychotherapeutic Centre
IRB: Institutional Review Board
OPD: Outpatient Department
PTSD: Posttraumatic Stress Disorder
SUD: Substance Use Disorders
